# The emergence and use of expanded carrier screening in gamete donation: A new form of repro‐genetic selection

**DOI:** 10.1111/bioe.13349

**Published:** 2024-08-31

**Authors:** Nicky Hudson, Cathy Herbrand, Lorraine Culley

**Affiliations:** ^1^ Centre for Reproduction Research De Montfort University Leicester UK

**Keywords:** fertility, gamete donation, genetic inheritance, genetic screening, IVF, risk

## Abstract

With the continued expansion and commercialisation of fertility treatments, the selection and matching of donors have become more sophisticated and technologised. As part of this landscape, new form of genetic screening: ‘expanded carrier screening’ (ECS) is being offered as a technique to avoid the risk of donors passing on genetic conditions to future offspring. Allowing donors to be tested for hundreds of genetic conditions simultaneously, ECS marks a considerable departure from traditional ‘family history’ models of screening, which rely on an individual's knowledge of family health. There is growing evidence of a drive towards the use of ECS within the fertility sector, and a growing number of clinics are offering it for a fee, as part of an egg or sperm donation cycle or as an add‐on to IVF treatment. In this article, we use methods of critical reflection to synthesise data from two studies to explore how ECS is being used to avoid genetic risk in IVF treatment using donor gametes. We suggest that ECS is a new form of repro‐genetic selection—a selective reproductive technology—with specific and important implications for donors, recipients and clinicians and with the potential to reconfigure the scope and application of gamete donation. We examine these implications and conclude that the existing policy blind spot relating to this development in fertility treatment practice needs to be urgently addressed.

## 1 INTRODUCTION

Reproductive donation (using eggs and sperm) usually involves some level of ‘matching’ of donors with recipients, as people seek to create families where parents and children share characteristics. Historically, this has been largely related to the selection of donors with particular physical characteristics, interests, or aptitudes that correspond, on some level, with those of recipients.^1^ With the continued expansion and commercialisation of fertility treatments, the selection and matching of donors has become more sophisticated and technologised. Historically, the process of donor selection involves the medical screening of candidates in order to identify and possibly screen out individuals with particular diseases (e.g., HIV) or genetic disorders. In the case of screening for genetic conditions, traditionally this has involved a family history questionnaire to identify if a donor is at risk of passing on a particular disorder to future offspring, either because of family history or because of membership of an ethnic group with a high prevalence of a particular condition (e.g., Tay Sachs amongst the Ashkenazi Jewish population). In this existing model of screening, if a potential risk is identified, this might be confirmed with traditional carrier testing, which involves testing the donor for that specific condition. However, this method cannot always guarantee identification of at‐risk individuals, not least since it relies on donors having access to family health history. A handful of high‐profile cases have highlighted scenarios in which serious genetic conditions have been discovered in offspring after egg or sperm donation.^2^ In such cases, it appears a significant pathogenic variant has been inadvertently passed from a donor to the person conceived via those gametes. Whilst rare, these cases illustrate the potential for genetic risk to be a feature of donor IVF.

A new form of genetic screening: ‘expanded carrier screening’ (ECS) is being offered by some clinics as a technique to mitigate some of these risks in third‐party donation. This genome‐wide technology allows identification of carriers of recessive autosomal conditions (i.e., those inherited via transmission of an altered copy of the gene from both parents) or X‐linked conditions (inherited via a faulty gene on the X chromosome), regardless of family history, ethnicity, or other risk factors. The tests are described as potentially revolutionary, in that an individual egg or sperm provider can be tested for hundreds of possible disease‐causing variants simultaneously, rather than testing for a single condition following traditional history‐taking. As a consequence, ECS has the potential to be used as a first‐line tool to detect a large range of recessive disorders individuals may be at risk of transmitting. Beyond its use in gamete donation, ECS can also be used to test any heterosexual couples who wish to know if they are at risk of transmitting a recessive disorder to their future children. This technology is therefore increasingly offered by fertility or genetic clinics as a ‘compatibility test’, and in some countries is part of national healthcare programmes to provide information and choices to prospective parents.^3^

There is growing evidence of a drive towards the use of ECS within the fertility sector as an increasing number of panels (a testing format where a specific group of genes are tested simultaneously) are developed and a growing number of clinics are offering it as part of an egg or sperm donation cycle or as an add‐on to IVF treatment more generally.^4^

There is also growing evidence that clinics are using the results of these tests to advertise donors to recipient clients in new ways, by framing certain donors as offering a risk‐free conception, a healthy pregnancy, or a good ‘genetic match’ for recipients.^5^ The introduction of this technology is expanding existing methods of selection and matching and raising new questions about how clinics and fertility professionals can and should embed this into treatment packages.

As a highly novel intervention, the screening of donors using next‐generation sequencing also raises a number of important questions for social theorists, ethicists, and policy makers. A key part of this debate is that, in many contexts, regulation and professional guidance on screening donors do not yet cover these techniques. For example, within Europe, the EU Tissue and Cells Directive (pan‐European regulation that concerns how human tissue of all kinds can be procured and managed) currently sets out guidance for how gamete providers should be screened within its member states. However, this guidance was published in 2004, prior to the development and roll out of ECS.^6^ With the development of newer methods, a vacuum has been created in the guidance around such tests and what they mean for the landscape of reproductive genetics, understandings about risk and inheritance, the provision of genetic counselling for donors and recipients and any impacts on their future reproductive‐decision making, and implications for the practice of fertility clinics and the wider industry.

Authors have highlighted the increasing diversification of IVF and the ‘parcelling out’ of the reproductive process into ‘ever more specialized moments of sequencing, editing, sorting, selecting, diagnosing, discarding and terminating’.^7^ As an example of this parcelling out, in this article, we consider how ECS for gamete donors (hereafter ‘ECS‐GD’), represents a new, technologised form of selection—of certain kinds of donors—rendering it as an example of what Wahlberg and Gammeltoft refer to as a ‘selective reproductive technology’.^8^ They suggest that whilst technologies such as IVF are designed to seek a viable pregnancy and birth, *selective* reproductive technologies are designed to ‘determine pregnancy outcomes in very specific ways, which is to say by preventing or promoting the birth of specific kinds of children’.^9^ In this context, we suggest that ECS is a new repro‐genetic technology framed as a technique which can facilitate the birth of children without genetic conditions caused by recessive pathogenic variants, via the selection of particular donors. We use methods of critical reflection to explicate this argument,^10^ drawing on two large‐scale studies: one recently completed on gamete donation in Europe (the ‘EDNA’ study ESRC: ES/N010604/1), and a current study exploring the use of ECS for non‐at‐risk heterosexual couples (the ‘PRECAS’ study: ESRC: ES/W012456/1). We explore how ECS‐GD is emerging and being taken up by fertility clinics and how its implications might be reshaping the IVF landscape more broadly. We suggest that ECS‐GD has specific and important implications for donors, recipients, and clinicians which need to be urgently considered for regulation and practice.

## 2 THE EMERGENCE OF ECS IN GAMETE DONATION

Developed in the early 2000s, the test requires a saliva or blood sample and results can be generated in as little as two weeks, providing an overview of an individual's carrier status for a range of conditions. ECS has the potential to radically alter the screening and selection process for gamete donors because for the first time it allows clinics to test for multiple pathogenic variants which may not be known about by the prospective donor; a key advantage over the use of family history questionnaires. When used alongside existing methods of screening, ECS therefore has the potential to offer clinics greater flexibility and potential for widening the screening of donors. In allowing greater specificity of testing, ECS also offers the potential for new ways of managing donor selection, which has historically revolved around selection of physical (and sometimes psychological) features. Whilst these physical characteristics are often framed as inherited, they have not traditionally included health‐based, genetic features.

There appears to be two main approaches to the use of ECS‐GD emerging in fertility clinics. First, ECS‐GD may be used to facilitate the direct rejection of candidates who may transmit a severe disorder. For example, evidence from one study shows that out of 883 candidate donors, 17.6% were rejected based on their ECS result for 46/47 genes.^11^ In this model, any prospective donors identified as carrying pathogenic variants may be excluded at the point of recruitment. A second approach is to allow such individuals to join a pool of potential donors that can be selected from. Gamete recipients would then be tested to ensure they are free from the pathogenic variant(s) carried by the selected donor before using this donor. Allowing donors to remain in the pool regardless of carrier status is potentially an appealing approach for clinics because it avoids rejection of a large number of possible donors.^12^ Leaving carriers in the pool means moving from a model of screening out, to one of selective matching by integrating principles of what has been referred to as ‘genetic compatibility’ into criteria used to find the ‘perfect match’ for each recipient. Using AI (artificial intelligence) software, new platforms are emerging which are able to use ECS results along with other selection criteria such as facial matching methods (e.g., ‘Perfect Match 360°’ or ‘Fenomatch’), to ensure a match between donor and recipient.

Despite its enthusiastic uptake in some contexts, ECS does not completely eradicate risks of transmission.^13^ While ECS can test for a number of recessive and X‐linked disorders, not all possible disease‐causing variants are known or can be tested for. As a result, there will always remain a risk of transmitting a genetic disorder caused by a variant which hasn't yet been identified. Moreover, the person conceived from fertility treatment may also develop a genetic disorder caused by a ‘de novo’ change (i.e., a spontaneous variant that has not been inherited from parents) or multiple genes, which can't be detected by ECS. Finally, it is also possible that recipients may still decide to use a donor who has been identified as carrying a particular mutation whilst not being tested themselves; thereby accepting the risk of possible transmission.

Evidence suggests that to date, uptake of ECS by fertility clinics has been most rapid in the United States,^14^ where donors are plentiful and treatment is already highly commercialised. However, our research in Europe highlights the emergence and growth of the use of ECS in this context, where historically fertility treatment has tended to be less commercialised (with public funding for treatment available in several countries) and where shared regulatory guidance exists with regard to management of donated human tissue, including screening for donors.^15^ However, even within Europe, wide variation exists. In some countries with a relatively large number of donors, for example, egg donors in Spain or sperm donors in Denmark, an increasing number of clinics and gamete banks now routinely screen donors^16^ and many advertise this on their websites. In countries where donors are in relatively short supply, the United Kingdom for instance, there has been lower uptake of ECS‐GD as this reduces the (already small) donor pool if donors are ruled out for certain recipients or screened out altogether. However, even in countries with lower uptake, new dilemmas exist for clinics. For example, if clinics are importing gametes (from overseas), or procuring them from a commercial bank, and they have been screened with ECS and pathogenic variants identified, clinicians are faced with decisions about whether to inform recipients about this risk and offering genetic counselling; to provide a ‘matching test’; ignore this information; or reject use of these gametes altogether. The complexity of ECS and its implications therefore extends beyond clinics where it is routinely used.

Because ECS‐GD offers new ways to select, stratify, and manage donors, it presents a potentially significant shift in clinical and commercial activity. From our analysis, we have identified four themes which demonstrate key implications of this development and which we suggest need urgent consideration: the imperative to create healthy pregnancies; new forms of genetic responsibilisation; the saleability of ‘good genes’; and the impact on donors’ health and reproductive decision‐making. Following discussion of these themes, we suggest that ECS‐GD can be conceptualised as a selective reproductive technology. We conclude by suggesting that ECS has the potential to shift the logic of gamete donation from overcoming infertility to the conception of a child without any genetic disorders.

## 3 THE IMPLICATIONS OF ECS IN GAMETE DONATION

### 3.1 An imperative to create healthy pregnancies

Whilst avoiding the transmission of a known or likely disorder was an expectation of existing screening practices in gamete donation, this process was perceived as leaving room for error. ECS‐GD, on the other hand, is being positioned as a tool which can deliberately prevent the transmission of potential genetic disorders to the future child via new modes of selection and matching. Since ECS offers the possibility to prevent inheritance of many genetic disorders, it simultaneously creates and reinforces an expectation that having a healthy child is now an accessible, operational, and saleable possibility through third‐party donation. It creates an expectation that gamete donation is, in part, a process that can facilitate the conception of healthy pregnancies (and children) through the selection of donors with ‘good genes’. In centreing processes of genetic selection and an imperative to create optimised or ‘healthy’ pregnancies, ECS‐GD therefore also generates and amplifies new responsibilities for the avoidance of genetic diseases. In this sense, it intersects with on‐going debates about other forms of reproductive screening which, it has been argued, may engender pressure to screen in the interests of avoiding disability or illness.^17^ It may also create unrealistic expectations for recipients regarding the health of future children; particularly given ECS won't completely eradicate other types of genetic condition (or other kinds of health problems). As well as contributing to a more generalised imperative to optimise conception and offspring, individual recipients might feel ‘judged’ by clinicians (or others) if they refuse to be tested for an identified condition or to select another donor.

### 3.2 ECS and new forms of genetic responsibilisation

Positioned as a new technology of detection and prevention, the development and use of ECS‐GD in a practical sense has implications for ideas about what has been referred to elsewhere as ‘genetic responsibility’;^18^ with the potential for clinicians, recipients, and donors to be enroled in novel processes of selection in the quest for healthy offspring.

For fertility clinicians, there may be a shift in responsibility towards matters of ‘genetic health’ as they may be leading decisions about testing, which conditions to test for, which panels to use, and offers of counselling. These new responsibilities have been discussed in cases where a disorder has been transmitted to a donor‐conceived child. For example, in a recent Spanish case, a clinic was ordered by the Court of Valencia to pay 40,000 euros to the parents of a child born with a genetic condition for not using all technologies available to limit such risk.^19^ Such cases potentially imply that clinicians should proactively seek to identify all risks related to inherited disorders and mitigate them if they have the means to do so, and may, in part, account for the growth of ECS‐GD in the fertility sector. There are also decisions about which conditions people are tested for, for example, whether this includes a small number of well‐known but severe conditions or an extensive panel with a wide range of conditions varying in potential severity.^20^ It remains unclear what happens and where responsibility is seen to lie if a recessive disorder is passed to a child in a clinic offering ‘screened’ donors.

It could be argued that ECS‐GD has the potential to position gamete recipients as potentially responsible for transmission of a genetic condition, and therefore also responsible for avoiding such risk. Those seeking fertility treatment are usually keen to avoid any potential risks and may therefore be willing to pay for ECS as part of their treatment accordingly. ECS therefore has the potential to responsibilise recipient parents in new ways; by prioritising ideas of selection and genetic health and in the process, potentially increasing the geneticisation of fertility treatment more generally. In this context, understandings of IVF success, inheritance, and health are increasingly framed via the lens of genomics.

The question of where this responsibility resides in ECS‐GD may depend on who makes decisions about testing.^21^ For example, it raises questions about whether both the donor and the gamete‐providing intended parent be tested (and matched) or only the donor (who is then either selected or rejected); to what extent any testing should be opt‐in or opt‐out for recipients, and whose responsibility these decisions are.

Where testing is decided on by recipients, this may be based on whether (1) they can afford the test and (2) they themselves want to be screened (to check they can use their selected donor). While a U.S. survey showed that many intending recipients (91%) would prefer having donors tested with ECS rather than traditional family/ancestry history,^22^ that might not materialise in practice if patients have to pay. Other studies highlight the impact of cost on decision‐making.^23^ Recipients are therefore placed at a complex intersection of decisions related to their own treatment, the testing of donors, additional costs, and implications for any future offspring.

In terms of donors, it appears that they have little—and perhaps ironically the least—autonomy in decision‐making around ECS, other than to decide whether or not to donate in a clinic which mandates ECS testing for donors. Our research highlights that in fact donors are not always clear about the kinds of testing and screening that has been performed, whether this includes ECS or traditional screening.^24^ Depending on how ECS is used in clinics, there is a possibility that it works to reduce the responsibilities of donors—removing their agency in the process. However, if they donate in clinics where they are obliged to actively participate in screening, including ECS, it potentially increases their responsibilities by enroling them in new forms of health citizenship, whereby they are positioned as genetically responsible for the health of offspring they help to create.^25^ Existing research suggests that donors already feel a sense of obligation around being a ‘good donor’;^26^ ECS has the potential to expand and amplify this sense of responsibility. It is not currently clear how much information donors are being given about this shift in practice.

### 3.3 The saleability of ‘good genes’: ECS as a treatment ‘add‐on’

With the move towards more tailored selection and the ability to match donors and recipients, on the basis of carrier status, comes the potential for genetic inheritance to become a saleable feature of the treatment landscape. Whilst the direct purchase of gametes in Europe is illegal, a range of practices are pushing the *process* of donor selection further into the marketplace. Unlike traditional forms of donor selection, which are based on phenotypical characteristics such as skin, hair, and eye colour and carried out by clinic staff as part of the process, ECS‐GD is a distinct technique charged for by the clinic in addition to standard cycle costs. The cost of screening is folded into treatment packages; driving up the cost of a cycle (e.g., the addition of ECS may increase a cycle of egg donation treatment by £1000–2000). In this case, the object for sale is a form of selectivity specifically related to genetic inheritance, which is separated out and made purchasable. The product becomes the possibility or choice to more directly control genetic inheritance, which is advertised to patients via clinic websites (e.g., the European Sperm Bank indicates that a test it offers, GeneXmatch, is ‘a screening test that improves the chances of your future baby being *healthy’*).

Previous research, including our own, exploring donor marketing has considered how imaginaries of ‘good genes’ are drawn on in the advertising of donors.^27^ These studies demonstrate how in traditional donor selection the good genes narrative has been employed with reference to desirable physical characteristics or personality traits. In ECS‐GD, in addition to physical traits, donors (and their gametes) are marketed as being specifically disease or risk‐free by virtue of their ECS results, which may position them as free of specific pathogenic variants, or as described above, free of variants of concern for particular recipient/s. ECS means that donors can be placed into new hierarchies based more explicitly around genetic profiles, with clinics able to market donors in new ways. By doing so, ECS creates the promise that clinics will help patients find more than just a ‘healthy donor’ but the ‘perfect‐for‐you donor’, including at the genetic level.

Given its focus on the prediction of genetic risk in donor populations, ECS‐GD routinises and centres practices of selection—already a well‐developed feature of donation^28^—with the process of selection amplified by the use of highly specialised tools, and significantly, novel commercial choreographies. This shift brings with it the possibility for new and varying levels of demand for particular donors and the possibility of varied pricing structures for treatment with the gametes from those donors.^29^ These new commercialised practices have the potential to catalyse new forms of reproductive tourism to places like the United States and Spain: already buoyant markets for novel repro‐interventions and which are becoming hubs for ECS. ECS can therefore be thought of as a very particular form of treatment ‘add‐on’; which builds on current markets and practices, but which centres genetic inheritance as a saleable feature of gamete donation. Here, intended parents are in effect purchasing the possibility to select a donor with the ‘right’ genetic profile when they include ECS as an add‐on to their donation treatment cycle.^30^

### 3.4 Impact on donors’ own health and reproductive decision‐making

In the context of gamete donation, ECS raises a new set of questions in relation to the experiences and meaning‐making of the donor who may experience selection and de‐selection against new criteria with potential (future) implications for their own health and/or reproductive decision‐making. The use of ECS for gamete donors raises questions about how (or if), in the context of a positive result for a specific disease‐causing variant, complex genetic health‐related information will be communicated to donors and how they will make sense of these data. It raises questions about whether treatment/test providers will fully explain the process and implications for donors’ own health.^31^ Genetic information can be complex to understand and takes time to explain. Current professional and ethical debate has highlighted the increased need for genetic counselling implied by ECS, whereby donors who are identified as carrying a pathogenic variant may need specialist counselling.^32^ It is not currently clear how practice is emerging in this regard and how, in a commercially oriented setting, adequate opportunities for counselling will be provided.

One unpublished study from the United States explored the experience of egg donors and found that their experience of receiving feedback and counselling about the results of their screening was of mixed quality.^33^ Some donors were not told results and some only received results through a portal, rather than via a health care professional or counsellor. They also found there was less disclosure of results to donors by egg banks as compared to clinics. This raises questions relating to ‘ownership’ of information about a donor's test result, which may be considered linked to whoever is requesting or paying for the test. In the U.S. study, donors experienced refusals when requesting information about results or were ‘locked out’ of information provision, for example, by clinics asking them to pay for the test if they then withdrew from the process.

If donors are found to be carriers of a genetic condition, this raises questions for their own future reproductive decision‐making.^34^ A positive test could, for example, have a negative impact on the donor's sense of themselves as healthy or ‘fit’ for reproduction.^35^ In the United States, donors had mixed feelings about the significance of results, with some downplaying the importance; for example, when results related to very rare conditions. However, it could lead to later complexity if donors themselves decide to conceive and feel they need to ask their partner to be tested to assess their shared risk, or if they need to have pre‐natal screening.^36^ Knowing about carrier status has the potential to change, in new and currently uncertain ways, how future personal reproductive decisions are approached by donors; something to be seen as the technology rolls out further.

These concerns raise the question of whether ECS may consist of a form of ‘overtreatment’ for donors. The concept of overtreatment refers to ‘the consumption of health care that brings the risk of harm for little or no appreciable benefit’.^37^ Questions about overtreatment of donors exist more generally; since (as is the case with other kinds of tissue donation), they are subject to a wide range of medical and/or invasive interventions not of direct benefit to them as individuals. Egg donation in particular is a highly invasive procedure and concerns have been expressed about, for example, the impact of powerful hormonal drugs and the process of egg retrieval on so‐called ‘non‐patient’ donors.^38^ ECS‐GD presents new questions about overtreatment of donors since it involves application of additional medical interventions not designed to specifically benefit the donor. In the context of gamete donation, ECS may represent a form of what has been referred to as ‘over diagnosis’ – that is, a diagnosis that may be ‘“correct” according to current standards but the diagnosis or associated treatment has a low probability of benefitting the patient, and may instead be harmful’.^39^ The introduction of new kinds of testing or the use of screening for reassurance purposes have both been highlighted as forms of over diagnosis.^40^ ECS‐GD potentially fits this conceptualisation, in that it is a form of testing which is used, not to diagnose illness but to identify carrier status. Whilst in this context the screening is designed to identify risk of carrier status amongst donors, this information is not used to inform decision‐making for the donor (though this may be a corollary) but to improve outcomes for recipients.

## 4 DISCUSSION: ECS AS A NEW FORM OF REPRO‐GENETIC SELECTION

We suggest that the specific features and implications of ECS‐GD outlined in this article make it a new form of ‘selective reproductive technology’.^41^ Wahlberg and Gammeltoft suggest that whilst technologies such as IVF are designed to seek a viable pregnancy and birth, *selective* reproductive technologies are designed to ‘determine pregnancy outcomes in very specific ways, which is to say by preventing or promoting the birth of specific kinds of children’.^42^ A potential shift in the logic of reproductive technologies from successful pregnancies to healthy babies^43^ contributes to the production of wider imaginaries of the ‘well‐born’,^44^ and to an on‐going desire to avoid ‘imperfect pregnancies’.^45^ This shift builds on and extends earlier considerations about the use of reproductive technologies. For example, in her work on egg donation, Martin explains, ‘the decision to parent always involves risk, whether that is through “natural” or assisted conception, or by fostering/adoption. SRTs, such as third‐party egg transfer, sperm sorting and PGD (pre‐implantation genetic diagnosis), however, may be used to mitigate risks and give intended parents some modicum of control over the “product” by assessing, ranking, and selecting gametes and embryos’.^46^

We suggest that ECS‐GD is a distinctive and novel form of repro‐genetic selection because it pushes the use of genetic selection in the quest for a healthy baby much further. First, unlike PGD and NIPT (non‐invasive pre‐natal testing) which are currently mainly used to diagnose a specific condition or a limited number of chromosomal anomalies, ECS allows screening for hundreds of pathogenic variants simultaneously, increasing significantly awareness and identification of transmission risks. Second, it creates new forms of reproductive and genetic matching. A new mode of ‘ECS‐enabled matching’ consists of unselecting a donor carrying the same pathogenic variant as the recipient. In this respect, ‘matching’ goes beyond physical resemblance, instead becoming a way to circumvent the intended parents’ genetics and eliminating risk of transmission. ECS‐GD therefore expands and amplifies existing logics of ‘suitability’ in gamete donation by increasing the ways in which donors can be selected and matched to recipients. In doing so, it creates a new market for the selection of good genes and genetic inheritance, increasing stratification, and allowing clinics that offer screening to profit from additional treatment revenue. Third, and perhaps most significantly, in pushing genetic testing and selection further upstream, ECS‐GD removes the need to test and potentially discard the embryo or foetus—as in the case of prenatal or pre‐implantation testing—and therefore presents a potentially less controversial or morally challenging option to prospective parents. Finally, we suggest that ECS‐GD is a novel form of repro‐genetic selection since it permits the widening of the parameters in which screening takes place. It achieves this by moving clinics away from the practice of focused screening of otherwise at‐risk individuals and towards the ‘broad spectrum’ screening of low‐risk groups. ECS‐GC may therefore be reflective of an increasing geneticisation of pre‐conception and fertility treatments more generally, which is moving ever‐closer towards a model of fully personalised care. In this respect, ECS appears to be part of the growing commercialisation of both genetic technologies (as seen with techniques like genealogical testing) and reproductive technologies more generally. As the fertility industry expands its reach into new jurisdictions and new markets and in the process attracts new investors, novel sources of diversification and associated income become a central driver of activity and innovation. This expansion of the broader fertility marketplace is also explanatory for how and why ECS has potentially undergone rapid uptake in the field of IVF specifically (when compared, for example, to other areas of genetic medicine): existing commercialised pathways in IVF have facilitated uptake of ECS‐GD and provided the context for its growth.

## 5 CONCLUSION

Whilst reproductive technologies such as IVF were first developed as a means to overcome involuntary childlessness for heterosexual couples, their reach and scope has evolved to include a wide range of techniques, applications, and users. The increasing diversification of IVF and the ‘parcelling out’ of the reproductive process has resulted in ever more distinct, discrete, and specialised moments of activity.^47^ Within this context, the landscape of reproductive genetics has grown exponentially and ECS is one example of this development. ECS presents a number of specific implications when utilised in the context of gamete donation, including new practical conundrums for practitioners and recipients, the disruption of existing treatment pathways, new questions regarding information giving and care of donors, and the amplification of ideas about ‘good genes’. In this sense, ECS potentially transforms the aim and significance of medical screening and selection in reproductive donation, and in doing so it creates the perception that ‘good (donor) genes’ equal ‘good (child) health’, without dilemmas about discarding embryos or terminating pregnancies.

We suggest that the sociotechnical, policy, and commercial interactions shaping the emergence of ECS are giving rise to new sets of priorities, practices, and professional rationalities in the context of assisted reproductive technology, many of which are emerging without regulation and policy oversight. In order to address this emerging ‘blind spot’, we recommend both a broad (social) and specific (practice) debate about the significance of ECS in gamete donation and its implications. If it is decided that the benefits of ECS outweigh its harm or limitations, one might want to consider how to harmonise practices; for instance, by offering ECS more widely and providing a clear and consistent set of recommendations for clinicians, recipients, and donors. Professionals would also need more certainty about whether carrier‐positive donors can be used or not, and in which circumstances, and what their responsibilities are in the case of the possible transmission of a disorder. Recipients could be systematically informed of the possibility of using ECS, what benefits (or not) it provides, and what their options, responsibilities, and subsequent costs would be. They should also have the possibility to refuse the test and for their choice not to be motivated only by financial considerations. Finally, prospective gamete donors need to be given full information about the use of ECS‐GD, including the possibility to access their results, when presenting at clinics.

## Footnotes

1 Homanen, R. (2018). Reproducing whiteness and enacting kin in the Nordic context of transnational egg donation: Matching donors with cross‐border traveller recipients in Finland. *Social Science and Medicine*, *203*, 28–34; Rubin, L., de Melo‐Martin, I., Rosenwaks, Z., & Cholst, I. N. (2015). Once you're choosing, nobody's perfect: Is more information necessarily better in oocyte donor selection? *Reproductive BioMedicine Online*, *30*(3), 311–318.

2 NBC NEWS. (2009). *Sperm donor passed on deadly heart defect*. Retrieved from Sperm donor passed on deadly heart defect (nbcnews.com); Sheldon, T. (2002). Children at risk after sperm donor develops late onset genetic disease. *BMJ: British Medical Journal*, *324*(7338), 631.

3 ECS can therefore theoretically be used to screen any couples in a broader population or within communities where there is a higher risk for recessive disorders to be transmitted, for example, in the context of first‐cousin marriage. These broader potential uses are not the focus of this article.

4 Markets and Markets. (2019). *Carrier screening markets*. https://www.marketsandmarkets.com/Market-Reports/carrier-screening-market-72642285.html

5 Isley, L., Hornberger, K., Seitz, S., Baldwin, K., Lockwood, K., & Awwad, R. (2023). Diagnoses of autosomal recessive conditions in donor‐conceived offspring in the context of carrier screening practices: A collaboration between three large U.S. sperm banks. *Fertility & Sterility*, *120*(4), Supplement e92.

6 Official Journal of the European Union. (2004). *Directive 2004/23/EC of the European Parliament and of the Council of 31 March 2004 on setting standards of quality and safety for the donation, procurement, testing, processing, preservation, storage and distribution of human tissues and cells*. Retrieved from 30707 48.48 (europa.eu).

7 Wahlberg, A., Dong, D., Song, P., & Jianfeng, Z. (2021). The platforming of human embryo editing: Prospecting “disease free” futures. *New Genetics and Society*, *40*(4), 367–383.

8 Wahlberg, A., & Gammeltoft, T. (2018). *Selective reproduction in the 21st century*. Springer International Publishing.

9 Ibid: 5–6.

10 Hauskeller, C., & Beltrame L. (2016). Hybrid practices in cord blood banking. Rethinking the commodification of human tissues in the bioeconomy. *New Genetics and Society*, *35*(3), 228–245.

11 Payne, M. R., Skytte, A. B., & Harper, J. C. (2021). The use of expanded carrier screening of gamete donors. *Human Reproduction*, *36*(6), 1702–1710.

12 Boada, M., Abulí, A., Clua, E., Palacios, G., Veiga, A., Armengol, L., Estivill, X., Coroleu, B., & Barri, P. N. (2017). Genetic matching between recipients and oocyte donors. *Current Trends in Clinical Embryology*, *4*, 52.

13 2% of couples have matching genetic variants: 1 in 4 of their children will have the condition. See Henneman, L., Borry, P., Chokoshvili, D., Cornel, M. C., van El, C. G., Forzano, F., Hall, A., Howard, H. C., Janssens, S., Kayserili, H., Lakeman, P., Lucassen, A., Metcalfe, S. A., Vidmar, L., de Wert, G., Dondorp, W. J., & Peterlin, B. (2016). Responsible implementation of expanded carrier screening. *European Journal of Human Genetics*, *24*, e1–e12.

14 Markets and Markets, op. cit. note 4.

15 Hudson, N., Culley, L., Herbrand, C., Pavone, V., Pennings, G., Provoost, V., & Lafuente Funes, S. (2020). Reframing egg donation in Europe: New regulatory challenges for a shifting landscape. *Health Policy and Technology*, *9*(3), 308–313.

16 Abulí, A., Boada, M., Rodríguez‐Santiago, B., Coroleu, B., Veiga, A., Armengol, L., Barri, P. N., Pérez‐Jurado, L. A., & Estivill, X. (2016). NGS‐based assay for the identification of individuals carrying recessive genetic mutations in reproductive medicine. *Human Mutation*, *37*(6), 516–523.

17 Thomas, G. M., Rothman, B. K., Strange, H., & Latimer, J. E. (2021). Testing times: The social life of non‐invasive prenatal testing. *Science, Technology and Society, 26*(1), 81–97.

18 Rose, N., & Novas, C. (2004). Biological citizenship. In: A. Ong & S. Collier (Eds.), *Global assemblages: Technology, politics, and ethics as anthropological problems* (pp. 439–463). Blackwell Publishing.

19 Pavone, V., & Turrini, M. (2023, November). *A perfect match. Routinizing genetic matching (GM) in Spanish assisted reproduction*. Mapping the genetic screening between public health and personalised medicine. Symposium, Madrid.

20 Mertes, H., Lindheim, S. R., & Pennings, G. (2018). Ethical quandaries around expanded carrier screening in third‐party reproduction. *Fertility and Sterility*, *109*(2), 190–194.

21 Ibid.

22 Jackson, E., Edwards, J., Besser, A., & Isley, L. J. (2017). Recipients’ perspectives regarding expanded carrier screening of gamete donors. *Fertility and Sterility*, *108*(3), e265–e266.

23 Robson, S. J., Caramins, M., Saad, M., & Suthers, G. (2020). Socioeconomic status and uptake of reproductive carrier screening in Australia. *Australian and New Zealand Journal of Obstetrics and Gynaecology, 60*(6), 976–979.

24 Hudson, N., Coveney, K., & Herbrand, C. (2018, July). *Genetics, heritability and family histories: materialising the healthy egg donor in clinical screening practices*. EASST Conference. Lancaster.

25 Hudson, N. (2023, April). *The goose with the golden eggs: On scarcity, altruism and health citizenship in the new egg bioeconomy*. British Sociological Association Annual Conference, Stream Plenary, Manchester.

26 Nordqvist, P., & Gilman, L. (2022). *Donors: Curious connections in donor conception*. Emerald Publishing.

27 Almeling, R. (2007). Selling genes, selling gender: Egg agencies, sperm banks, and the medical market in genetic material. *American Sociological Review*, *72*(3), 319–340; Coveney, C., Hudson, N., Lafuente‐Funes, S., Jacxsens, L., & Provoost, V. (2022). From scarcity to sisterhood: The framing of egg donation on fertility clinic websites in the UK, Belgium and Spain. *Social Science & Medicine*, *296*, 114785.

28 Martin, L. J. (2018). They don't just take a random egg: egg selection in the United States. In A. Wahlberg & T. Gammeltoft (Eds.), *Selective reproduction in the 21st century* (pp. 151–171). Springer International Publishing.

29 Pennings, G. (2020). Expanded carrier screening should not be mandatory for gamete donors. *Human Reproduction*, *35*(6), 1256–1261.

30 Mertes, H., et al., op. cit. note 20.

31 Ibid.

32 Ibid.

33 Miller, E. (2022). *Understanding oocyte donor perceptions of expanded carrier screening and genetic testing* [Unpublished masters thesis]. Retrieved from OhioLINK ETD: Miller, Elianna Brittany.

34 Mertes, H., et al., op. cit. note 20; Miller, op. cit. note 33.

35 Mertes, H., et al., op cit. note 20; Chokoshvili, D., Van Steijvoort, E., & Borry, P. (2018). Ethical challenges in offering expanded carrier screening in the context of third‐party reproduction. *Medecine de la Reproduction, 20*(4), 334–339.

36 Mertes, H., et al., op. cit. note 20.

37 Hensher, M., Tisdell, J., & Zimitat, C. (2017). “Too much medicine”: Insights and explanations from economic theory and research. *Social Science & Medicine*, *176*, 77–84.

38 Tober, D. M., Richter, K., Zubizarreta, D., & Daneshmand, S. (2023) Egg donor self‐reports of ovarian hyperstimulation syndrome: Severity by trigger type, oocytes retrieved, and prior history. *Journal of Assisted Reproduction and Genetics*, *40*, 1291–1304.

39 Armstrong, N. (2018). Overdiagnosis and overtreatment as a quality problem: Insights from healthcare improvement research. *BMJ Quality Safety*, *27*(7), 571–575.

40 Armstrong, N. (2021). Overdiagnosis and overtreatment: a sociological perspective on tackling a contemporary healthcare issue. *Sociology of Health & Illness*, *43*(1), 58–64.

41 Wahlberg & Gammeltoft, op. cit. note 8, pp. 5–6.

42 Ibid.

43 Ehrich, K., & Williams, C. (2010). A ‘healthy baby’: The double imperative of preimplantation genetic diagnosis. *Health*, *14*(1), 41–56.

44 Raz, A. (2009) Eugenic utopias/dystopias, reprogenetics, and community genetics. *Sociology of Health and Illness*, *31*(4), 602–616.

45 Löwy, I. (2017). *Imperfect pregnancies: A history of birth defects and prenatal diagnosis*. JHU Press.

46 Martin, op. cit. note 28, p. 152.

47 Wahlberg, A., et al., op. cit. note 7.

